# Endothelial-Myocardial Angiocrine Signaling in Heart Development

**DOI:** 10.3389/fcell.2021.697130

**Published:** 2021-07-01

**Authors:** Hyeonyu Kim, Mingqiang Wang, David T. Paik

**Affiliations:** ^1^Stanford Cardiovascular Institute, Stanford University, Stanford, CA, United States; ^2^Department of Medicine, Division of Cardiovascular Medicine, Stanford University School of Medicine, Stanford, CA, United States

**Keywords:** endothelial cell, cardiac development, single-cell sequencing, myocardial compaction, cardiomyocyte

## Abstract

Vascular endothelial cells are a multifunctional cell type with organotypic specificity in their function and structure. In this review, we discuss various subpopulations of endothelial cells in the mammalian heart, which spatiotemporally regulate critical cellular and molecular processes of heart development via unique sets of angiocrine signaling pathways. In particular, elucidation of intercellular communication among the functional cell types in the developing heart has recently been accelerated by the use of single-cell sequencing. Specifically, we overview the heterogeneic nature of cardiac endothelial cells and their contribution to heart tube and chamber formation, myocardial trabeculation and compaction, and endocardial cushion and valve formation via angiocrine pathways.

## Introduction

Vascular endothelial cells, which compose the innermost lining of blood vessels, are a multifunctional cell type that dynamically governs tissue function and homeostasis by regulating blood flow, vascular tone, oxygen and nutrient transport, inflammation, and delivery of plasma-borne macromolecules (Aird, 2007; Ricard et al., 2021). Contingent upon the type of tissue or the organ of residence, endothelial cells exhibit a significant level of cellular, molecular, and functional heterogeneity (Augustin and Koh, 2017). Moreover, these organ-specific roles of the endothelial cells are manifested by the unique gene expression signatures that correspond to the organotypic characteristics of the local tissue (Ricard et al., 2021). Recent advances in single-cell sequencing techniques have particularly been advantageous in accelerating our understanding of the organotypic specificity of endothelial cells, as they have been able to eschew the critical limitations of primary endothelial cell culture where removal of endothelial cells from their local microenvironment rapidly promotes endothelial-to-mesenchymal transition (EndoMT) and thereby alters the identity and function of the isolated endothelial cells (Jakab and Augustin, 2020; Paik et al., 2020a). For example, single-cell RNA-sequencing of endothelial cells isolated from 11 major tissues of adult mice led to the profiling of transcriptomic features of quiescent arterial, venous, capillary, and lymphatic endothelial cells (Kalucka et al., 2020), and similarly an organism-wide analysis of *Tabula Muris* dataset unveiled novel markers of organ- and sex-specific endothelial cells in major organs of adult mice (Paik et al., 2020b). Notably, these single-cell transcriptome studies employed predictive analysis of intercellular communication, whereby unique sets of angiocrine (i.e., secreted) factors from endothelial cells were identified in each of the organs investigated.

In this review, we focus on the angiocrine role of heart-specific endothelial cells in cardiac development and the pathological conditions that arise from dysregulation of the cardiac endothelium. Mammalian heart development encompasses coordinated, spatiotemporal interactions of several cell types in a highly orchestrated manner. Consequently, dysregulation in the intercellular communication or improper generation of any one of the cell types can lead to congenital heart defects and pathological phenotypes (Daniela et al., 2010), which we discuss in this review.

## Heterogeneity of Cardiac Endothelial Cells

In the mammalian heart, endothelial cells are the most abundant cell type next to cardiomyocytes and cardiac fibroblasts, occupying approximately 12 and 8% in cell number in atria and ventricles, respectively (Litviňuková et al., 2020). The precise role of cardiac endothelial cells in cardiac development varies by the specialized function of their subpopulations. Traditionally, pan-endothelial cells in the heart have been identified by the expression of cluster of differentiation 31 (CD31), CD34, CD105 (endoglin), CD144 (vascular endothelial cadherin), CD309 (vascular endothelial growth factor receptor 2), and endothelial nitric oxide synthase (Pratumvinit et al., 2013). Subpopulations of cardiac endothelial cells are classified based on their spatial location in the heart and their gene expression profile (Chen et al., 2014). Endocardial cells for example are specialized endothelial cells unique to the developing heart, which act as a barrier between blood and myocardium in the innermost part of the heart tissue. Endocardial cells are identified by expression of nuclear factor in activated T-cell, cytoplasmic 1 (Nfatc1; de la Pompa et al., 1998; Ranger et al., 1998), natriuretic peptide receptor 3 (Npr3; Hui et al., 2016), and cytokine-like protein 1 (Cytl1; Feng et al., 2019). Coronary endothelial cells expressing apelin (Apln; Tian et al., 2014), fatty acid-binding protein 4 (Fabp4; He et al., 2014), and CD36 (Feng et al., 2019) create and maintain coronary vessels within the myocardium, providing oxygen and nutrients to parenchymal cells and removing waste products. In addition to these two cell types, endothelial cells of the aorta have been shown to express EH domain-containing 3 and family with sequence similarity 167 member B (Fam167b; Feng et al., 2019). Finally, lymphatic endothelial cells that exist in a relatively small number in the heart are marked by the expression of podoplanin (Pdpn; Cimini et al., 2019).

## Role of Endothelial Cells in Heart Tube and Chamber Formation

**FIGURE 1:**
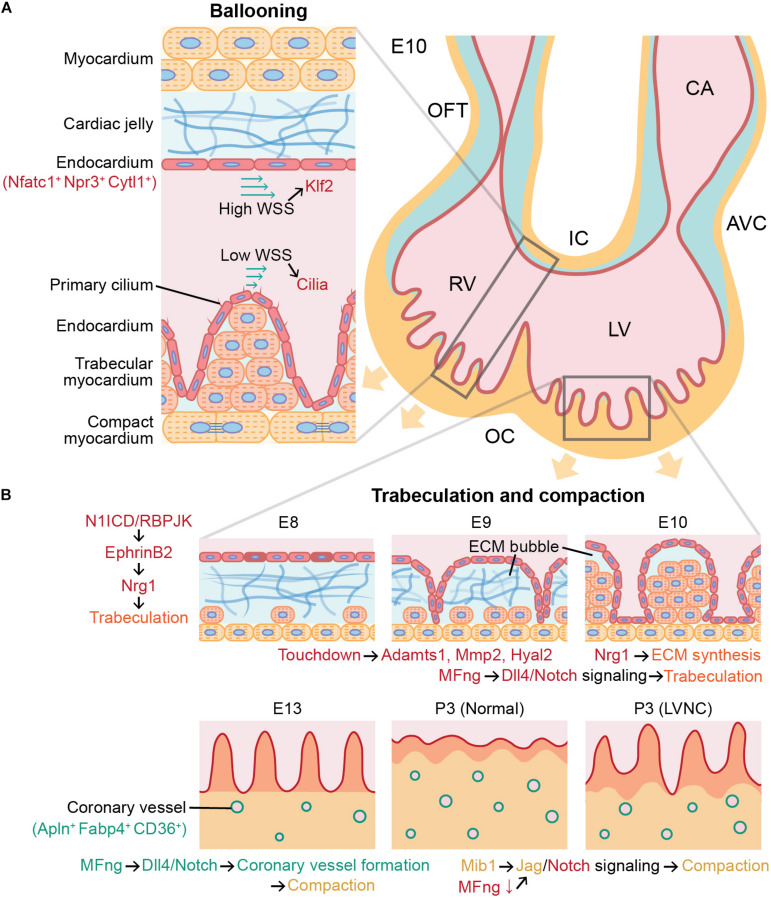
Role of angiocrine signaling in cardiac chamber ballooning, trabeculation, and compaction during cardiac development. Angiocrine signaling of cardiac endothelial cells governs major cellular and molecular processes in mammalian heart development. **(A)** Ballooning is achieved by more active proliferation in the outer curvature (OC) than in the inner curvature (IC), and this asymmetric proliferation between the inner and outer curvatures is associated with Klf2 and cilia signaling in endocardial cells activated by wall shear stress (WSS). **(B)** Initiation of trabeculation and growth of trabeculae (orange) in ECM bubble are regulated by Nrg1 secreted by endocardial cells (red). After trabeculation, compaction requires angiocrine signaling in both coronary endothelial cells (green) and endocardial cells (red). Endothelial dysfunction can cause left ventricular noncompaction (LVNC). Trabecular and compact cardiomyocytes are indicated in orange and yellow, respectively. E10, mouse embryonic day 10; OFT, outflow tract; RV, right ventricle; LV, left ventricle; CA, common atrium; AVC, atrioventricular canal; ECM, extracellular matrix; and P3, postnatal day 3.

During cardiac development, cardiac progenitors from the mesodermal primitive streak differentiate into a cardiac crescent, then form a linear heart tube (Clowes et al., 2014). The heart tube has the endocardium as the inner layer and the myocardium as the outer layer, and these two layers are separated by an acellular extracellular matrix (ECM) layer called the cardiac jelly (Eisenberg and Markwald, 1995). Using lineage tracing in mice, endocardium was reported to arise from a precardiac progenitor in the late primitive streak expressing fetal liver kinase 1 (Flk1; Ema et al., 2006; Harris and Black, 2010). Vascular endothelial cells derived from myocardial lineages in the second heart field progenitors were also found to partially contribute to endocardium formation (Verzi et al., 2005; Milgrom-Hoffman et al., 2011). Once the linear heart tube elongates, it undergoes dextral looping and forms chambers through ballooning. During the cardiac chamber ballooning, proliferation of cardiomyocytes is favored in the outer curvature of the heart tube than in the inner curvature, and it has been suggested that this asymmetrical formation of the heart tube is mediated by mechanotransduction via Krüppel-like factor 2 (Klf2). Hence, greater wall shear stress is applied by blood flow on the endocardium in the inner curvature than on the cells in the outer curvature (Figure 1A), which further activates Klf2 that functions as a sensor of fluid shear stress through cilia, Polycystins 2, and Trpv4 (Heckel et al., 2015). Expression of Klf2 gene subsequently activates Notch signaling (Li et al., 2020), and it renders a central role in EndoMT in the ventral atrioventricular endocardial cushion (Chang et al., 2011) and the inner curvature (Camenisch et al., 2010). However, more monocilia are present in the outer curvature than the inner curvature (Van der Heiden et al., 2006; Hierck et al., 2008), and hence it has been speculated that Flf2 activation in the inner curvature is mediated by ion channels instead of cilia. However, more detailed mechanisms of the effects of lower shear stress and more cilia in the outer curvature on cardiac chamber ballooning by the enhanced proliferation of cardiomyocytes are to be elucidated.

## Trabeculation and Compaction of Myocardium

Trabeculae are bundles of cardiomyocytes protruding from the inner wall of the ventricular chamber. The function of trabeculae is to rapidly increase the number of cardiomyocytes in the ventricular chamber and to facilitate oxygen and nutrient exchange in the myocardium via diffusion with their greater surface area prior to the vascularization by the coronary endothelial cells (Sedmera et al., 2000; Pérez-Pomares and de la Pompa, 2011). Trabeculation, or the formation of trabeculae, is initiated as the endocardial endothelial cells lining the inside of the heart tube penetrate the cardiac jelly in the ventricular outer curvature (Männer and Yelbuz, 2019). In mouse embryos, endocardial cells penetrated the cardiac jelly to reach the myocardium after Notch signaling was activated in the endocardium of the ventricular chamber (Grego-Bessa et al., 2007). As endocardial ridges are generated between the touchdowns between the endocardium and myocardium, a layer of the cardiac jelly becomes ECM bubbles (Figure 1B). Subsequently, the trabecular cardiomyocytes enclosed in the ECM bubble start to proliferate, and the trabecular unit grows long toward the ventricular lumen (del Monte-Nieto et al., 2018).

The ECM in the ECM bubble almost disappears as the ECM proteolytic genes ADAM metallopeptidase 1 (Adamts1), matrix metallopeptidase 2 (Mmp2), and hyaluronidase 2 (Hyal2) are activated in the endocardium. During this process, Notch1 intracellular domain (N1ICD)/RBPJK induces the secretion of neuregulin 1 (Nrg1) by activating EphrinB2 in endocardial cells (Grego-Bessa et al., 2007; Figure 1B). Nrg1 plays an important role in the growth of trabeculae through paracrine signaling by promoting proliferation of trabecular cardiomyocytes through ErbB2/4 in the myocardium (Liu et al., 2010). This was confirmed by Nrg1, ErbB2, and ErbB4 null mice that did not form trabeculae in ventricular chambers (Yarden and Sliwkowski, 2001). In addition, Nrg1 promotes ECM synthesis and formation of apical ECM bubbles required for rearrangement and growth of trabeculae (del Monte-Nieto et al., 2018), and the trabeculation was poorly developed in the Notch1 mutants (Grego-Bessa et al., 2007). The ECM secretion by endothelial cells during trabeculation has been confirmed by the single-cell RNA sequencing results of E9.5 and E11.5 mouse heart undergoing trabeculation that showed the high expression of hyaluronan and proteoglycan link protein 1 (Hapln1), a key regulator in developmental ECM interactions in ventricular endothelial cells (DeLaughter et al., 2016).

Moreover, bone morphogenetic protein 10 (Bmp10) is highly expressed in trabecular myocardium and is known to play an important role in trabeculae growth but not in the initiation of trabeculation (Chen et al., 2004). Activation of Bmp10 is also independent of EphrinB2 and Nrg1 expression during trabeculation (Grego-Bessa et al., 2007). Following the trabeculae growth, hypertrabeculation is prevented by endocardial Tie2 expression, which inhibits proliferation of cardiomyocytes (Qu et al., 2019). Single-cell analysis of the ligand-receptor pairs between cardiomyocytes and endocardial cells in E10.5 mouse heart also showed that expression of transforming growth factor beta 1 (Tgfb1), previously characterized to inhibit proliferation of cardiomyocytes, was specifically identified in endocardial cells, and expression of its receptors Tgfbr1 and Tgfbr3 was found in cardiomyocytes (Li et al., 2019). Together, these findings therefore indicate that endocardial cells play a major role in the formation of trabeculae by dynamically regulating promotion and inhibition of cardiomyocyte proliferation and ECM degradation.

At 6–7 weeks of gestation in human and E13.5 in mice, these trabecular structures undergo a compaction process that thickens the ventricular myocardium and smoothens the endocardial surface (Sedmera et al., 2000; MacGrogan et al., 2018). At this time, the myocardium can be divided into trabecular myocardium expressing Nppa in the inner part of the ventricular myocardium and compact myocardium expressing Hey2 in the outer part (Tian et al., 2017). When the myocardium becomes thicker than the diffusion limit due to the rapid proliferation of cardiomyocytes, a hypoxic environment facilitates the formation of coronary vessels in the compact myocardium for the exchange of oxygen and nutrients (Tian et al., 2014; MacGrogan et al., 2018). Using lineage-tracing and clonal analysis tools, it has been revealed that the origin of most coronary endothelial cells is pan-endocardium (Wu et al., 2012; Hui et al., 2018), and about 10% of them are from proepicardium (Katz et al., 2012; Cano et al., 2016). This compaction is continued until postnatal day 28 in mice (Tian et al., 2017), and while some coronary endothelial cells of the postnatal heart are not expanded from embryonic coronary endothelial cells but instead converted from ventricular endocardial cells in the inner part of the myocardium (Tian et al., 2014). Similar to trabeculation, compaction is also largely influenced by endothelial cells through paracrine signaling, and defects in this process can lead to cardiomyopathies such as in the form of left ventricular noncompaction (Wengrofsky et al., 2019).

In autocrine and paracrine signaling during heart development, the Fringe family of glycosyltransferases attaches to the Notch and contributes to ligand selectivity (Panin et al., 1997). In particular, β-1,3-N-Acetylglucosaminyltransferase manic fringe (MFng) modulates the spatiotemporal specificity of Notch-receptor interactions by enhancing Dll4-Notch1 signaling and diminishing Jag-Notch signaling through glycosylation (Panin et al., 1997; Yang et al., 2004; D’Amato et al., 2016b). Expression of MFng is promoted in endocardial during trabeculation, but is required to be down-regulated during compaction to activate myocardial Jag1 and Jag2 signaling to Notch1. In addition to inactivation of MFng, activation of Mib1 gene up-regulates Jag1 in compact cardiomyocytes (D’Amato et al., 2016a). Since Jag1 expression is important for compaction, inactivation of Mib1 causes abnormally thin compact myocardium and large noncompacted trabeculae in mice (Luxán et al., 2013). On the other hand, activation of Dll4 by MFng activation in coronary endothelial cells is required for coronary vessel formation, which is needed for proper myocardial compaction (D’Amato et al., 2016b; Rhee et al., 2018). Therefore, MFng, which promotes Dll4-Notch signaling, must be activated or inactivated depending on the specific type of cardiac endothelial cells for the correct compaction process. In addition, deletion of Jarid2 in both coronary endothelial and endocardial cells increases the methylation at the Notch1 (Mysliwiec et al., 2012) and leads to noncompaction and hypertrabeculation of ventricular myocardium (Mysliwiec et al., 2011).

Compact cardiomyocytes are known to proliferate faster than trabecular cardiomyocytes, which is associated with myocardial growth supported by coronary endothelial cells independent of a blood flow (Giordano et al., 2001; Rhee et al., 2018). These studies show that both endocardial and coronary endothelial cells can impact myocardial compaction via angiocrine pathways by regulating proliferation and cellular maturation of cardiomyocytes. The signaling pathways involved in trabeculation and compaction have been elucidated in *in vivo* models of mice, chicken, and zebrafish, but the detailed mechanisms of spatially restricted cue from the myocardium for the initiation of endocardial cell sprouting and angiocrine signaling between compact cardiomyocytes and coronary endothelial cells are not yet defined.

## Endocardial Cushion and Valve Formation

**FIGURE 2:**
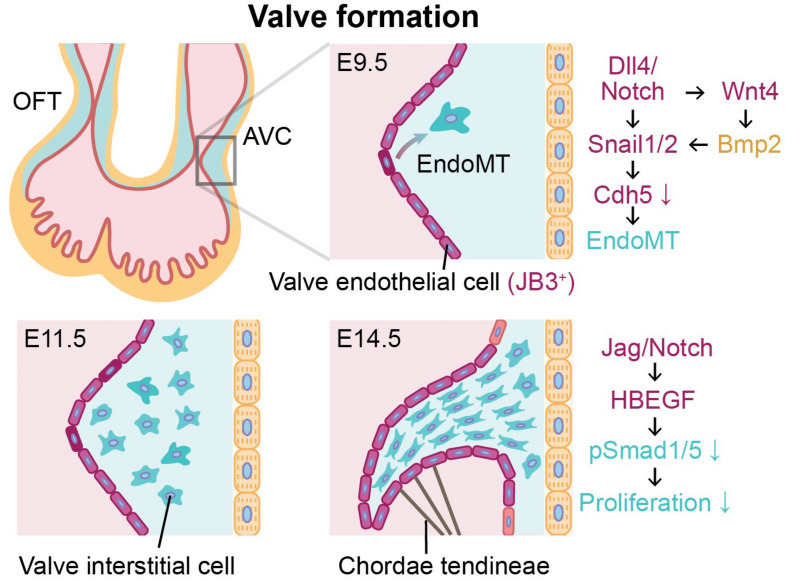
Endothelial-to-mesenchymal transition (EndoMT) of valve endothelial cells expressing JB3 and angiocrine signaling during valve formation between E9.5 and E14.5. Valve formation is initiated by EndoMT of valve endothelial cells (purple) to become valve interstitial cells (blue) in cardiac jelly. During valve formation, the cushion is remodeled and elongated by valve interstitial cells, and the mature valve leaflet is supported by chordae tendineae. Endocardial cells and cardiomyocytes are indicated in red and yellow, respectively. OFT, outflow tract; AVC, atrioventricular canal; E9.5, E11.5, E14.5, mouse embryonic day 9.5, 11.5, 14.5, respectively.

Following the looping of the heart tube, endocardial cushions are formed by expanding the cardiac jelly between the endocardium and the myocardium (Homan et al., 2019). In the embryonic heart, the endocardial cushions at the atrioventricular canal (AVC) and outflow tract (OFT) develop into atrioventricular (mitral/tricuspid) valves and semilunar (aortic/pulmonic) valves, respectively (Lin et al., 2012). When the valve formation is initiated, valve endothelial cells, a subpopulation of endocardium expressing JB3 (Wunsch et al., 1994) and lining the AVC and OFT, exhibit activated Dll4-Notch signaling (Figure 2). In the AVC, endocardial Notch1 signaling activates Wnt4 to induce Bmp2 expression in the adjacent myocardium (Wang et al., 2013). Mouse model studies in loss-of-function or gain-of-function of Notch1 and Bmp2 confirmed that expression of both Bmp2 in the AVC myocardium and N1ICD in the endocardium are required to induce EndoMT (Kisanuki et al., 2001; Luna-Zurita et al., 2010; Papoutsi et al., 2018). Notch activation also triggers upregulation of Snail1 and Snail2 expression (Donal et al., 2016) known to inhibit VE-cadherin (Cdh5) whose role is to regulate cell adhesion and cell-to-cell interactions of endocardial cells (Timmerman et al., 2004). Thus, down-regulation of Cdh5 reduces cell-cell contacts between endocardial cells and activates EndoMT. In addition to Notch signaling, Bmp2 has been shown to dramatically up-regulate Snail1 and Snail2 while down-regulating Slug in valve endothelial cells (Niessen et al., 2008). This subsequently leads to decreased expression of Cdh1 and Cdh5, which then promotes EndoMT (Niessen et al., 2008; Kroepil et al., 2012). In addition, expression of Slug in valve endothelial cells induces their migration by repressing VE-cadherin. Therefore, Snail and Slug regulate the initiation of cardiac cushion cellularization by inducing EndoMT (Niessen et al., 2008). Inactivation of Bmp receptor Alk2 in endothelial cells on the other hand results in cardiac cushion EndoMT defects from reduced expression of Snail, but not Slug and Hey2 (Niessen et al., 2008).

Recently, single-cell transcriptomic analysis of valve endothelial cells in the transition state demonstrated that EndoMT was related to PI3K-Akt signaling pathway and AGE-RAGE signaling (Kang et al., 2020). These endocardial-derived valve interstitial cells (VICs) invade the cardiac jelly, proliferate, and remodel the endocardial cushions to form thin elongate valve leaflets (Xiong et al., 2012). With cellular trajectory analysis from single-cell RNA-seq of aortic valve leaflets in calcific aortic valve disease (CAVD) patients, it has been reported that valve endothelial cells may participate in the calcification and thickening of aortic valve disease though more active EndoMT, demonstrated by greater expressions of secreted protein acidic and rich in cysteine and mesenchymal markers (Col1A1 and Cnn1) in transformed valve endothelial cells of CAVD patients than healthy controls (Kang et al., 2020). In the later stages of valve formation, activation of Jag1-Notch1 signaling causes increased Hbegf expression in the endocardium. This inhibits Bmp-phosphorylated Smad1/5 (p-Smad1/5) signaling to restrict proliferation of VICs in cardiac jelly (Donal et al., 2016). Moreover, Jag1 mutants resulted in dysmorphic and thicker valve leaflets due to uncontrolled proliferation of the VICs (Donal et al., 2016). These studies indicate that the investigation of paracrine signaling between the valve endothelial cells and the myocardium should be performed in a spatiotemporally controlled manner to obtain organized and well-functioning heart valves. As the majority of these findings to date have been obtained from animal models, future studies using human induced pluripotent stem cell or 3-dimensional *in vitro* tissue engineering models for embryonic human heart combining with single-cell analysis will be necessary to corroborate and to translate to human cardiac development and disease mechanisms.

## Conclusion

Angiocrine signaling of cardiac endothelial cells dynamically regulates proliferation and cellular maturation of cardiomyocytes and formation of endocardial cushion during mammalian heart development, playing a critical role in all phases of cardiogenesis. In this review, we described the angiocrine role of subpopulations of cardiac endothelial cells, including endocardial cells, coronary endothelial cells, and valve endothelial cells during cardiac development reported to date. Nonetheless, the heterogeneic nature of angiocrine signaling within the cardiac arterial, venous, and lymphatic endothelial cells remains poorly understood. Utilizing single-cell omics tools will enable profiling of specialized cell populations and heterogeneity with prediction of intercellular communication, further advancing our understanding of angiocrine function of endothelial cells in cardiac development, homeostasis, and disease. In addition, ongoing clinical trials will reveal whether therapeutics developed based on endocardial-myocardial angiocrine signaling will be effective in combatting human cardiovascular disease, as certain small-molecule candidates associated with nitric oxide-mediated and neuregulin-mediated pathways have been reported to demonstrate a reduction in mortality and improved cardiac output in heart failure patients in early phase clinical trials (Lim et al., 2015). Consequently, thorough elucidation of the role of angiocrine signaling in congenital heart disease will shed light on developing therapeutic strategies using genetic interventions.

## Author Contributions

HK wrote and revised the manuscript and generated the figures. MW and DP wrote, revised, and approved the submitted version of the manuscript. All authors contributed to the article and approved the submitted version.

## Conflict of Interest

The authors declare that the research was conducted in the absence of any commercial or financial relationships that could be construed as a potential conflict of interest.

## References

[B1] Aird W. C. (2007). Phenotypic heterogeneity of the endothelium. *Circ. Res.* 100 158–173. 10.1161/01.RES.0000255691.76142.4a 17272818

[B2] Augustin H. G. Koh G. Y. (2017). Organotypic vasculature: from descriptive heterogeneity to functional pathophysiology. *Science* 357:eaal2379. 10.1126/science.aal2379 28775214

[B3] Camenisch T. D. Runyan R. B. Markwald R. R. (2010). “Chapter 6.1–Molecular regulation of cushion morphogenesis,” in *Heart Development and Regeneration*, eds Rosenthal N. Harvey R. (Bostonm MA: Academic Press), 363–387. 10.1016/B978-0-12-381332-9.00018-9

[B4] Cano E. Carmona R. Ruiz-Villalba A. Rojas A. Chau Y.-Y. Wagner K. D. (2016). Extracardiac septum transversum/proepicardial endothelial cells pattern embryonic coronary arterio–venous connections. *Proc. Natl. Acad. Sci. U.S.A.* 113 656–661. 10.1073/pnas.1509834113 26739565 PMC4725486

[B5] Chang A. C. Y. Fu Y. Garside V. C. Niessen K. Chang L. Fuller M. (2011). Notch initiates the endothelial-to-mesenchymal transition in the atrioventricular canal through autocrine activation of soluble guanylyl cyclase. *Dev. Cell* 21 288–300. 10.1016/j.devcel.2011.06.022 21839921

[B6] Chen H. Shi S. Acosta L. Li W. Lu J. Bao S. (2004). BMP10 is essential for maintaining cardiac growth during murine cardiogenesis. *Development* 131 2219–2231. 10.1242/dev.01094 15073151 PMC2628765

[B7] Chen H. I. Sharma B. Akerberg B. N. Numi H. J. Kivelä R. Saharinen P. (2014). The sinus venosus contributes to coronary vasculature through VEGFC-stimulated angiogenesis. *Development* 141 4500–4512. 10.1242/dev.113639 25377552 PMC4302936

[B8] Cimini M. Garikipati V. N. S. de Lucia C. Cheng Z. Wang C. Truongcao M. M. (2019). Podoplanin neutralization improves cardiac remodeling and function after myocardial infarction. *JCI Insight* 4:e126967. 10.1172/jci.insight.126967 31287805 PMC6693826

[B9] Clowes C. Boylan M. G. S. Ridge L. A. Barnes E. Wright J. A. Hentges K. E. (2014). The functional diversity of essential genes required for mammalian cardiac development. *Genesis* 52 713–737. 10.1002/dvg.22794 24866031 PMC4141749

[B10] D’Amato G. Luxán G. de la Pompa J. L. (2016a). Notch signalling in ventricular chamber development and cardiomyopathy. *FEBS J.* 283 4223–4237. 10.1111/febs.13773 27260948

[B11] D’Amato G. Luxán G. del Monte-Nieto G. Martínez-Poveda B. Torroja C. Walter W. (2016b). Sequential Notch activation regulates ventricular chamber development. *Nat. Cell Biol.* 18 7–20. 10.1038/ncb3280 26641715 PMC4816493

[B12] Daniela T. Giordano F. J. Michael S. (2010). Cell communications in the heart. *Circulation* 122 928–937. 10.1161/CIRCULATIONAHA.108.847731 20805439 PMC2941440

[B13] de la Pompa J. L. Timmerman L. A. Takimoto H. Yoshida H. Elia A. J. Samper E. (1998). Role of the NF-ATc transcription factor in morphogenesis of cardiac valves and septum. *Nature* 392 182–186. 10.1038/32419 9515963

[B14] del Monte-Nieto G. Ramialison M. Adam A. A. S. Wu B. Aharonov A. D’Uva G. (2018). Control of cardiac jelly dynamics by NOTCH1 and NRG1 defines the building plan for trabeculation. *Nature* 557 439–445. 10.1038/s41586-018-0110-6 29743679

[B15] DeLaughter D. M. Bick A. G. Wakimoto H. McKean D. Gorham J. M. Kathiriya I. S. (2016). Single-cell resolution of temporal gene expression during heart development. *Dev. Cell* 39 480–490. 10.1016/j.devcel.2016.10.001 27840107 PMC5198784

[B16] Donal M. Gaetano D. Stanislao T. Beatriz M.-P. Guillermo L. Gonzalo del M.-N. (2016). Sequential ligand-dependent Notch signaling activation regulates valve primordium formation and morphogenesis. *Circ. Res.* 118 1480–1497. 10.1161/CIRCRESAHA.115.308077 27056911

[B17] Eisenberg L. M. Markwald R. R. (1995). Molecular regulation of atrioventricular valvuloseptal morphogenesis. *Circ. Res.* 77 1–6. 10.1161/01.RES.77.1.1 7788867

[B18] Ema M. Takahashi S. Rossant J. (2006). Deletion of the selection cassette, but not cis-acting elements, in targeted Flk1-lacZ allele reveals Flk1 expression in multipotent mesodermal progenitors. *Blood* 107 111–117. 10.1182/blood-2005-05-1970 16166582

[B19] Feng W. Chen L. Nguyen P. K. Wu S. M. Li G. (2019). Single cell analysis of endothelial cells identified organ-specific molecular signatures and heart-specific cell populations and molecular features. *Front. Cardiovasc. Med.* 6:165. 10.3389/fcvm.2019.00165 31850371 PMC6901932

[B20] Giordano F. J. Gerber H.-P. Williams S.-P. VanBruggen N. Bunting S. Ruiz-Lozano P. (2001). A cardiac myocyte vascular endothelial growth factor paracrine pathway is required to maintain cardiac function. *Proc. Natl. Acad. Sci. U.S.A.* 98 5780–5785. 10.1073/pnas.091415198 11331753 PMC33290

[B21] Grego-Bessa J. Luna-Zurita L. del Monte G. Bolós V. Melgar P. Arandilla A. (2007). Notch signaling is essential for ventricular chamber development. *Dev. Cell* 12 415–429. 10.1016/j.devcel.2006.12.011 17336907 PMC2746361

[B22] Harris I. S. Black B. L. (2010). Development of the endocardium. *Pediatr. Cardiol.* 31 391–399. 10.1007/s00246-010-9642-8 20135106 PMC2836465

[B23] He L. Tian X. Zhang H. Wythe J. D. Zhou B. (2014). Fabp4-CreER lineage tracing revealstwo distinctive coronary vascular populations. *J. Cell. Mol. Med.* 18 2152–2156. 10.1111/jcmm.12415 25265869 PMC4224549

[B24] Heckel E. Boselli F. Roth S. Krudewig A. Belting H.-G. Charvin G. (2015). Oscillatory flow modulates mechanosensitive klf2a expression through trpv4 and trpp2 during heart valve development. *Curr. Biol.* 25 1354–1361. 10.1016/j.cub.2015.03.038 25959969

[B25] Hierck B. P. Van der Heiden K. Poelma C. Westerweel J. Poelmann R. E. (2008). Fluid shear stress and inner curvature remodeling of the embryonic heart. Choosing the right lane! *ScientificWorldJournal.* 8:939501. 10.1100/tsw.2008.42 18661046 PMC5849229

[B26] Homan K. A. Gupta N. Kroll K. T. Kolesky D. B. Skylar-Scott M. Miyoshi T. (2019). Flow-enhanced vascularization and maturation of kidney organoids in vitro. *Nat. Methods* 16 255–262. 10.1038/s41592-019-0325-y 30742039 PMC6488032

[B27] Hui Z. Lui K. O. Bin Z. (2018). Endocardial cell plasticity in cardiac development, diseases and regeneration. *Circ. Res.* 122 774–789. 10.1161/CIRCRESAHA.117.312136 29496799

[B28] Hui Z. Wenjuan P. Guang L. Xiuzhen H. Lingjuan H. Xueying T. (2016). Endocardium minimally contributes to coronary endothelium in the embryonic ventricular free walls. *Circ. Res.* 118 1880–1893. 10.1161/CIRCRESAHA.116.308749 27056912

[B29] Jakab M. Augustin H. G. (2020). Understanding angiodiversity: insights from single cell biology. *Development* 147:dev146621. 10.1242/dev.146621 32792338

[B30] Kalucka J. de Rooij L. P. M. H. Goveia J. Rohlenova K. Dumas S. J. Meta E. (2020). Single-cell transcriptome atlas of murine endothelial cells. *Cell* 180 764–779.e20. 10.1016/j.cell.2020.01.015 32059779

[B31] Kang X. Shangbo X. Yuming H. Tingwen Z. Ming L. Peng Z. (2020). Cell-type transcriptome atlas of human aortic valves reveal cell heterogeneity and endothelial to mesenchymal transition involved in calcific aortic valve disease. *Arterioscler. Thromb. Vasc. Biol.* 40 2910–2921. 10.1161/ATVBAHA.120.314789 33086873

[B32] Katz T. C. Singh M. K. Degenhardt K. Rivera-Feliciano J. Johnson R. L. Epstein J. A. (2012). Distinct compartments of the proepicardial organ give rise to coronary vascular endothelial cells. *Dev. Cell* 22 639–650. 10.1016/j.devcel.2012.01.012 22421048 PMC3306604

[B33] Kisanuki Y. Y. Hammer R. E. Miyazaki J. Williams S. C. Richardson J. A. Yanagisawa M. (2001). Tie2-cre transgenic mice: a new model for endothelial cell-lineage analysis in vivo. *Dev. Biol.* 230 230–242. 10.1006/dbio.2000.0106 11161575

[B34] Kroepil F. Fluegen G. Totikov Z. Baldus S. E. Vay C. Schauer M. (2012). Down-regulation of CDH1 is associated with expression of SNAI1 in colorectal adenomas. *PLoS One* 7:e46665. 10.1371/journal.pone.0046665 23029563 PMC3460919

[B35] Li G. Tian L. Goodyer W. Kort E. J. Buikema J. W. Xu A. (2019). Single cell expression analysis reveals anatomical and cell cycle-dependent transcriptional shifts during heart development. *Development* 146:dev173476. 10.1242/dev.173476 31142541 PMC6602356

[B36] Li X. Lu Q. Peng Y. Geng F. Shao X. Zhou H. (2020). Primary cilia mediate Klf2-dependant Notch activation in regenerating heart. *Protein Cell* 11 433–445. 10.1007/s13238-020-00695-w 32249387 PMC7251007

[B37] Lim S. L. Lam C. S. P. Segers V. F. M. Brutsaert D. L. De Keulenaer G. W. (2015). Cardiac endothelium–myocyte interaction: clinical opportunities for new heart failure therapies regardless of ejection fraction. *Eur. Heart J.* 36 2050–2060. 10.1093/eurheartj/ehv132 25911648

[B38] Lin C.-J. Lin C.-Y. Chen C.-H. Zhou B. Chang C.-P. (2012). Partitioning the heart: mechanisms of cardiac septation and valve development. *Development* 139 3277–3299. 10.1242/dev.063495 22912411 PMC3424040

[B39] Litviňuková M. Talavera-López C. Maatz H. Reichart D. Worth C. L. Lindberg E. L. (2020). Cells of the adult human heart. *Nature* 588 466–472. 10.1038/s41586-020-2797-4 32971526 PMC7681775

[B40] Liu J. Bressan M. Hassel D. Huisken J. Staudt D. Kikuchi K. (2010). A dual role for ErbB2 signaling in cardiac trabeculation. *Development* 137 3867–3875. 10.1242/dev.053736 20978078 PMC3049280

[B41] Luna-Zurita L. Prados B. Grego-Bessa J. Luxán G. del Monte G. Benguría A. (2010). Integration of a Notch-dependent mesenchymal gene program and Bmp2-driven cell invasiveness regulates murine cardiac valve formation. *J. Clin. Invest.* 120 3493–3507. 10.1172/JCI42666 20890042 PMC2947227

[B42] Luxán G. Casanova J. C. Martínez-Poveda B. Prados B. D’Amato G. MacGrogan D. (2013). Mutations in the NOTCH pathway regulator MIB1 cause left ventricular noncompaction cardiomyopathy. *Nat. Med.* 19 193–201. 10.1038/nm.3046 23314057

[B43] MacGrogan D. Münch J. de la Pompa J. L. (2018). Notch and interacting signalling pathways in cardiac development, disease, and regeneration. *Nat. Rev. Cardiol.* 15 685–704. 10.1038/s41569-018-0100-2 30287945

[B44] Männer J. Yelbuz T. M. (2019). Functional morphology of the cardiac jelly in the tubular heart of vertebrate embryos. *J. Cardiovasc. Dev. Dis.* 6:12. 10.3390/jcdd6010012 30818886 PMC6463132

[B45] Milgrom-Hoffman M. Harrelson Z. Ferrara N. Zelzer E. Evans S. M. Tzahor E. (2011). The heart endocardium is derived from vascular endothelial progenitors. *Development* 138 4777–4787. 10.1242/dev.061192 21989917 PMC3190386

[B46] Mysliwiec M. R. Bresnick E. H. Lee Y. (2011). Endothelial Jarid2/Jumonji is required for normal cardiac development and proper Notch1 expression. *J. Biol. Chem.* 286 17193–17204. 10.1074/jbc.M110.205146 21402699 PMC3089562

[B47] Mysliwiec M. R. Carlson C. D. Tietjen J. Hung H. Ansari A. Z. Lee Y. (2012). Jarid2 (Jumonji, AT rich interactive domain 2) regulates NOTCH1 expression via histone modification in the developing heart. *J. Biol. Chem.* 287 1235–1241. 10.1074/jbc.M111.315945 22110129 PMC3256911

[B48] Niessen K. Fu Y. Chang L. Hoodless P. A. McFadden D. Karsan A. (2008). Slug is a direct Notch target required for initiation of cardiac cushion cellularization. *J. Cell Biol.* 182 315–325. 10.1083/jcb.200710067 18663143 PMC2483533

[B49] Paik D. T. Cho S. Tian L. Chang H. Y. Wu J. C. (2020a). Single-cell RNA sequencing in cardiovascular development, disease and medicine. *Nat. Rev. Cardiol.* 17 457–473. 10.1038/s41569-020-0359-y 32231331 PMC7528042

[B50] Paik D. T. Lei T. Williams I. M. Siyeon R. Hao Z. Chun L. (2020b). Single-Cell RNA sequencing unveils unique transcriptomic signatures of organ-specific endothelial cells. *Circulation* 142 1848–1862. 10.1161/CIRCULATIONAHA.119.041433 32929989 PMC7658053

[B51] Panin V. M. Papayannopoulos V. Wilson R. Irvine K. D. (1997). Fringe modulates Notch–ligand interactions. *Nature* 387 908–912. 10.1038/43191 9202123

[B52] Papoutsi T. Luna-Zurita L. Prados B. Zaffran S. de la Pompa J. L. (2018). Bmp2 and Notch cooperate to pattern the embryonic endocardium. *Development* 145:dev163378. 10.1242/dev.163378 29853617

[B53] Pérez-Pomares J. M. de la Pompa J. L. (2011). Signaling during epicardium and coronary vessel development. *Circ. Res.* 109 1429–1442. 10.1161/CIRCRESAHA.111.245589 22158650

[B54] Pratumvinit B. Reesukumal K. Janebodin K. Ieronimakis N. Reyes M. (2013). Isolation, characterization, and transplantation of cardiac endothelial cells. *Biomed Res. Int.* 2013 359412. 10.1155/2013/359412 24282814 PMC3825130

[B55] Qu X. Harmelink C. Baldwin H. S. (2019). Tie2 regulates endocardial sprouting and myocardial trabeculation. *JCI Insight* 5:e96002. 10.1172/jci.insight.96002 31112136 PMC6629240

[B56] Ranger A. M. Grusby M. J. Hodge M. R. Gravallese E. M. de la Brousse F. C. Hoey T. (1998). The transcription factor NF-ATc is essential for cardiac valve formation. *Nature* 392 186–190. 10.1038/32426 9515964

[B57] Rhee S. Chung J. I. King D. A. D’amato G. Paik D. T. Duan A. (2018). Endothelial deletion of Ino80 disrupts coronary angiogenesis and causes congenital heart disease. *Nat. Commun.* 9:368. 10.1038/s41467-017-02796-3 29371594 PMC5785521

[B58] Ricard N. Bailly S. Guignabert C. Simons M. (2021). The quiescent endothelium: signalling pathways regulating organ-specific endothelial normalcy. *Nat. Rev. Cardiol.* 10.1038/s41569-021-00517-4 [Epub ahead of print]. 33627876 PMC7903932

[B59] Sedmera D. Pexieder T. Vuillemin M. Thompson R. P. Anderson R. H. (2000). Developmental patterning of the myocardium. *Anat. Rec.* 258 319–337. 10.1002/(SICI)1097-0185(20000401)258:4<319::AID-AR1<3.0.CO;2-O 10737851

[B60] Tian X. Hu T. Zhang H. He L. Huang X. Liu Q. (2014). De novo formation of a distinct coronary vascular population in neonatal heart. *Science* 345 90–94. 10.1126/science.1251487 24994653 PMC4275002

[B61] Tian X. Li Y. He L. Zhang H. Huang X. Liu Q. (2017). Identification of a hybrid myocardial zone in the mammalian heart after birth. *Nat. Commun.* 8:87. 10.1038/s41467-017-00118-1 28729659 PMC5519540

[B62] Timmerman L. A. Grego-Bessa J. Raya A. Bertrán E. Pérez-Pomares J. M. Díez J. (2004). Notch promotes epithelial-mesenchymal transition during cardiac development and oncogenic transformation. *Genes Dev.* 18 99–115. 10.1101/gad.276304 14701881 PMC314285

[B63] Van der Heiden K. Groenendijk B. C. W. Hierck B. P. Hogers B. Koerten H. K. Mommaas A. M. (2006). Monocilia on chicken embryonic endocardium in low shear stress areas. *Dev. Dyn.* 235 19–28. 10.1002/dvdy.20557 16145662

[B64] Verzi M. P. McCulley D. J. De Val S. Dodou E. Black B. L. (2005). The right ventricle, outflow tract, and ventricular septum comprise a restricted expression domain within the secondary/anterior heart field. *Dev. Biol.* 287 134–145. 10.1016/j.ydbio.2005.08.041 16188249

[B65] Wang Y. Wu B. Chamberlain A. A. Lui W. Koirala P. Susztak K. (2013). Endocardial to myocardial Notch-wnt-bmp axis regulates early heart valve development. *PLoS One* 8:e60244. 10.1371/journal.pone.0060244 23560082 PMC3613384

[B66] Wengrofsky P. Armenia C. Oleszak F. Kupferstein E. Rednam C. Mitre C. A. (2019). Left ventricular trabeculation and noncompaction cardiomyopathy: a review. *EC Clin. Exp. Anat.* 2 267–283.31799511

[B67] Wu B. Zhang Z. Lui W. Chen X. Wang Y. Chamberlain A. A. (2012). Endocardial cells form the coronary arteries by angiogenesis through myocardial-endocardial VEGF signaling. *Cell* 151 1083–1096. 10.1016/j.cell.2012.10.023 23178125 PMC3508471

[B68] Wunsch A. M. Little C. D. Markwald R. R. (1994). Cardiac endothelial heterogeneity defines valvular development as demonstrated by the diverse expression of JB3, an antigen of the endocardial cushion tissue. *Dev. Biol.* 165 585–601. 10.1006/dbio.1994.1278 7958424

[B69] Xiong Y. Zhou B. Chang C.-P. (2012). “Analysis of the endocardial-to-mesenchymal transformation of heart valve development by collagen gel culture assay,” in *Cardiovascular Development*, eds Peng X. Antonyak M. (Totowa, NJ: Humana Press), 101–109. 10.1007/978-1-61779-523-7_10 22222525

[B70] Yang L.-T. Nichols J. T. Yao C. Manilay J. O. Robey E. A. Weinmaster G. (2004). Fringe glycosyltransferases differentially modulate Notch1 proteolysis induced by Delta1 and Jagged1. *Mol. Biol. Cell* 16 927–942. 10.1091/mbc.e04-07-0614 15574878 PMC545923

[B71] Yarden Y. Sliwkowski M. X. (2001). Untangling the ErbB signalling network. *Nat. Rev. Mol. Cell Biol.* 2 127–137. 10.1038/35052073 11252954

